# Applying the 4Ps of social marketing to retain and engage participants in longitudinal cohort studies: generation 2 Raine study participant perspectives

**DOI:** 10.1186/s12874-022-01778-4

**Published:** 2022-11-05

**Authors:** Leesa Costello, Julie Dare, Manon Dontje, Claire Lambert, Leon Straker

**Affiliations:** 1 School of Medical and Health Sciences, Edith Cowan University, Joondalup, Western Australia; 2 Department of Rehabilitation, Physical Therapy Science and Sports, UMC Utrecht Brain Centre, University Medical Centre, Utrecht, the Netherlands; 3 Centre of Excellence for Rehabilitation Medicine, UMC Utrecht Brain Centre, University Medical Centre Utrecht, and De Hoogstraat Rehabilitation, Utrecht, the Netherlands; 4 School of Business and Law, Edith Cowan University, Joondalup, Western Australia; 5 School of Allied Health & enAble Institute, Faculty of Health Science, Curtin University, Perth, Western Australia

**Keywords:** The Raine study, Longitudinal cohorts, Retention, Attrition, Participant experience, Social marketing, Qualitative interviews, The 4Ps

## Abstract

**Background:**

Investigations of participant retention in longitudinal health and medical research, document  strategies that work best but overlook social marketing’s capacity to influence participant retention. After applying the social marketing framework: the idea that determining what longitudinal participants ‘buy’ (product), at what cost (price), in what location (place) and through which communication channels (promotion),  this paper  aims to inform and enhance retention efforts.

**Methods:**

This qualitative study was conducted through in-depth interviews with participants from the Raine Study that began in Western Australia in 1989. The Generation 2 participants, initially enrolled into the Raine Study as babies by their parents (Generation 1), are now young adults invited to attend follow-up studies and tests every few years. Our study defined ‘active’ participants (*n* = 17) as those who agreed to attend their 27 year follow-up, and ‘inactive’ (*n* = 12) participants as those who had attended neither of the past two follow-ups (22 and 27 years).

**Results:**

Raine Study participants experienced core, actual and augmented product benefits. Inactive participants focused on the costs (price) associated with participation, and were more likely to suggest tele-health (place) strategies to overcome barriers to follow-up attendance. Both active and inactive participants found professional processes and friendly staff made the Raine Study environment appealing, suggested that social media (promotion) was underutilised, and offered novel ideas to enhance engagement.

**Conclusions:**

Social marketing can support the development of differentiated strategies addressing the unique needs and wants of active and inactive participants. Sophisticated cohort segmentation can reach participants in a more meaningful way, reinforce the study ‘brand’ and guard against attrition.

**Supplementary Information:**

The online version contains supplementary material available at 10.1186/s12874-022-01778-4.

## Background

Longitudinal cohort studies contribute to understanding health and disease over the life-course and inform policy and practice [1, 2]. In successful longitudinal studies, investment of community and participant resources generates data from which discoveries can be made. Difficulties in retention threaten the quality of longitudinal studies due to insufficient sample size and subsequent loss of statistical power [3, 4], or selective retention that can bias estimates of associations [5, 6].

### Retention

Interest in strategies to promote retention has increased over the last two decades [7]. A systematic review of retention strategies in longitudinal cohort studies by Robinson et al. [7] identified 985 different retention strategies across 82 studies, with “contact and scheduling methods” and “visit characteristics” (p. 85) among the most common themes. Booker et al. found reminders demonstrated some benefit and monetary incentives improved retention [8]. A more in-depth investigation of strategies used by successful cohort studies with high retention rates (80%) by Abshire et al. [9] yielded findings that were similar to Robinson’s, with additional key findings providing important insights. Highly skilled and humane research staff and ‘personal touches’ such as the use of newsletters and birthday cards effectively fostered positive connections between participants. Successful cohort study managers adjusted retention strategies based on their expertise and experience, but did not always update the study protocols to reflect small changes. Teague and colleagues’ recent systematic review [10] of retention across 95 longitudinal cohort studies saw strategies focused on participant burden as the strongest predictors of improvements in retention, and recommended cohort managers use discretion when choosing strategies to resource. They identified 44 new or innovative strategies delivered through the internet and mobile devices.

Choi and colleagues’ found one of the most effective retention strategies was acknowledging the important contribution participants were making to the study, every time participants were contacted [11]. This strategy affirmed participants were more than “mere data points or passive subjects for the study, but invaluable collaborators of the study” ( [11] p. 301). Table 1 summarises the key categories of retention strategies discussed in the literature.

**Table 1:** Categories of retention strategies

Retention strategy category	Description	Booker C, et al. 2011 [8]	Robinson K, et al., 2015 [7]	Abshire M, et al., 2017 [9]	Teague S, et al., 2018 [10]
Contact and scheduling methods	A systematic method for participant contact, appointment scheduling, and cohort retention monitoring is used		✓	✓	
Visit characteristics	Minimize participant burden through characteristics and procedures of follow-up study clinic e.g., flexible appointments, convenient locations.		✓	✓	
Study personnel	Characteristics, training, and management of study personnel		✓	✓	
Nonfinancial incentives	Provide nonfinancial incentives or tokens of appreciation	✓	✓	✓	
Financial incentives	Provide financial incentives or payment	✓	✓	✓	
Reminders	Provide reminders about appointments and study participation	✓	✓	✓	✓
Special tracking methods	Methods of tracking hard-to-find or difficult participants		✓	✓	✓
Study description	Explain to participants the study requirements and details, including potential benefits and risks		✓	✓	
Benefits of study	Provide benefits to participants and families that are directly related to the nature of study		✓	✓	
Reimbursements	Provide reimbursement for research related expenses or tangible support to facilitate participation		✓	✓	
Study identity	Create study identity for participants		✓	✓	
Community involvement	Involve community in study design, recruitment, and retention		✓	✓	✓
Reducing barriers to participation	e.g., offering childcare services, assistance with transport and parking, utilising a participant sub sample to evaluate data collection approaches for the next wave				✓
Other methods	e.g., Methods of posting, personalised information letters, length of questionnaire (e.g., shorter)	✓			

### Social marketing framework

As Kotler, Lee and Rothschild (2006) observed, social marketing is “a process that applies marketing principles and techniques to create, communicate and deliver value in order to influence target audience behaviours that benefit society (e.g., public health, safety, the environment and communities) as well as the target audience” (cited in [12] p. 23). Despite recent reviews of retention strategies not specifically discussing marketing or acknowledging application of marketing techniques, an earlier study by Kobayashi et al. demonstrated that a social marketing approach can help attract research participants, while addressing issues around social exclusion and cohesion in hard-to-reach target groups [13]. This study argued that social marketing should inform all research designs, at least in terms of recruitment and engagement/retention.

Use of a social marketing framework should be based on a thorough understanding of the target audience’s perceptions of the benefits, barriers, motivations and influences. One of the longest running cohort studies of its kind [1, 2], the Raine Study, incorporates a life-course approach to understanding health and disease [1], and has the mission to “improve lifelong health and quality of life through ground-breaking, impactful research that examines influences, pathways and outcomes from before birth and throughout life’s course” [14]. The first paper of our two-paper series on this study [15], explained how active and inactive participants experienced their involvement, explored their perspectives on the benefits and barriers relating to their participation and highlighted the motivating factors and influencers on their decision to remain (or not remain) in the study. This second paper documents qualitative research findings and application of the ‘social marketing mix’ of the 4Ps – Product, Price, Place and Promotion [16], to understand factors related to retention and attrition from the perspective of active and inactive participants.

Social marketing involves a comprehensive use of segmentation, targeting and positioning strategies to induce positive behaviour [17], and we draw on Lee and Kotler’s 2016 [16] commentary here. *Segmentation* identifies subgroups or segments of a population with shared characteristics such as needs, wants, lifestyles, behaviour, and values (e.g., segmentation by generation in a cohort study) making them likely to respond similarly to the social marketing action. *Targeting* involves development of a uniquely appropriate social marketing program for the identified market segment (e.g., generational specific recruitment methods for a single generation of participants in cohort studies). Branding can help attain the desired *Positioning* of the product, enabling the target audience to ‘perceive’ the desired behavioural action being sought (e.g., participation in the cohort study and its individual and altruistic benefits) relative to competing behaviours.

#### The 4Ps

**Fig. 1:**
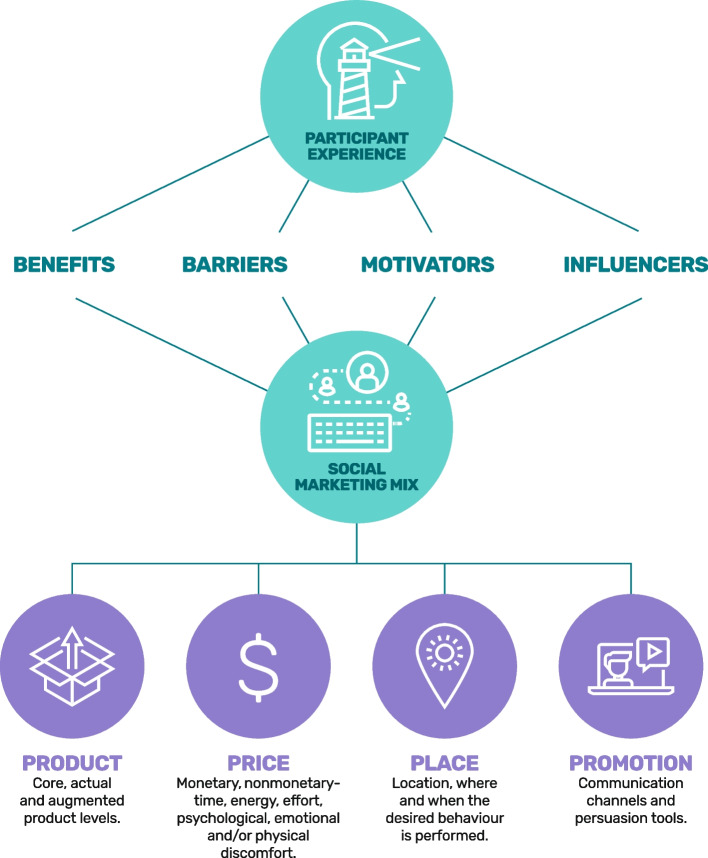
Participant experience and social marketing framework for cohort study participants

Lee and Kotler [16] described the discipline-specific components of the 4Ps (product, price, place, and promotion) comprising the dominant paradigm of the social marketing framework. *Product* denotes the set of benefits associated with the desired behaviour or service usage. The core product is the benefits the target market wants and expects in exchange for performing the desired behaviour. The actual product refers to the product features and its design, while the augmented product includes supplementary benefits or services that enhance the core product. *Price* is the sum of the costs (whether a monetary or nonmonetary exchange) the target market “pays” when adopting the desired behaviour. *Place* refers to where and when the target audience is required to engage in the behaviour, attain any associated goods, and receive any related services. *Promotion* is the persuasive communication used to stimulate action in the target audience. Fig. 1 represents the framework and indicates how the understanding phase – discussed in the first paper of this research [15] – led to a conceptualisation of the 4Ps.

In exploring the 4Ps of social marketing to better inform retention and engagement strategies in longitudinal studies, this second paper addresses the research questions: what **product** do Raine participants currently ‘buy’ and at what cost **(price)**; where do they have to go **(place)** to participate; and what communication channels are used to persuade them to act **(promotion)**? This paper also considers participants’ current perceptions of the Raine Study’s brand and provides recommendations to help ‘reposition’ the brand to strengthen engagement and retain participants.

## Methods

The methods used in this qualitative study are detailed in the initial paper [15]. The Raine Study began in 1989 with recruitment of 2900 pregnant women (Generation 1). The resultant 2868 births formed the Generation 2 cohort and this qualitative study involves the young adult Generation 2 participants who were invited to complete questionnaires and independent assessments when they were 20, 22, and 27 years old. These ongoing assessments include biomedical testing, varying in degree of invasiveness (e.g., measurements for height, weight, and blood pressure to provision of blood and urine samples) dependent on the aim and extent of health issues being explored [1, 2].

### Sample and recruitment

Participants for this study were recruited by Raine Study staff via telephone or email. As the study aimed to explore issues of retention using a social marketing framework, those staff purposely recruited ‘active’ participants who had agreed to attend their 27 year follow up, as well as ‘inactive’ participants who had not attended either of the past two follow-ups (22 and 27 years). Early in the recruitment process, researchers noticed an overrepresentation of female participants, and Raine Study staff were then requested to proactively contact male participants (specifically inactive males) so data saturation could be reliably determined. Recruitment continued until no new relevant information emerged during the final few interviews. In total, 29 participants were recruited (17 active and 12 inactive).

### Data collection

One focus group, and face-to-face and telephone interviews were conducted between September 2017 and February 2018 (by the first and second authors), incorporating interview prompts based on the social marketing framework (see appendix A in the Initial paper [15]). Individual interviews were undertaken as inactive participants were unwilling in a focus group setting to disclose why they had dropped out of the study. All inactive participants (n-12) opted for interviews via telephone, while all active participants (*n* = 17) opted to do face-to-face interviews. The focus group and face-to-face interviews were conducted at Raine Study House, the research facility used for previous follow-up visits.

Semi-structured interviews were used to acquire an understanding of the experience of being a Raine Study participant. Each interview lasted between 20 and 90 minutes and was digitally recorded. Participants were urged to describe their experiences of partaking in the Raine Study at different life stages, and how their expectations changed. In accordance with the social marketing theoretical framework, questions were purposely framed to examine the barriers, benefits, motivators, and others who might affect the participant’s tendency to attend follow-up assessments. Participants provided informed consent, and ethical approval was provided by Edith Cowan University’s Human Research Ethics Committee (approval number 18242). The interview guide (see Additional file 1) was created and evaluated by members of the research team and a panel of experts connected with the Raine Study.

Rapport building techniques were utilised as part of a larger group of techniques intended to establish trustworthiness and rigor in qualitative research [18]. To minimise social desirability bias, participants were urged to ‘have their say’ so the study could grasp their genuine, ‘behind the scenes’ experiences, and were advised that the interviewers were independent of the Raine Study, and their responses would be de-identified [15]. As the two interviewers were also aware of the potential for their personal views on the importance of retention in longitudinal research to influence how they interacted during the interviews, care was taken to avoid responses that participants could potentially interpret as judgmental.

Data saturation was reached when new insights were not revealed from interviews with both active and inactive participants. Given the demographic cohesiveness, data saturation was able to be achieved relatively quickly [19].

### Data analysis

While data was extracted from the same data set collected and reported on in our earlier paper on the Raine Study [15], this current paper interprets the data differently to explore the 4Ps of social marketing to inform retention and engagement strategies for longitudinal studies. Audio recordings were transcribed verbatim, and NVIVo 12 used to facilitate analysis.

Following Braun and Clarke [20] and Liamputtong [21], the analysis involved constant comparisons and team coding processes to build rigorous themes around the 4Ps of social marketing. Initial coding was conducted by the first author, and co-coding checks were carried out in collaboration with the second author.

## Results

Findings relevant to how active and inactive participants perceived the positioning and branding of the Raine Study provide ‘big picture’ insights about how they connect with and relate to the brand. Delving deeper into the 4Ps, the following section presents the themes and sub-themes for active versus inactive participants.

### Positioning and branding

Our investigation revealed distinct differences between how active and inactive participants positioned the Raine Study. Table 2 lists these themes.

**Table 2:** Positioning insights

Theme	Subtheme	Codes	
		***Active participants***	***Non-active participants***
**Positioning**	Connection to brand	Credibility Effort Longevity Raine family/cool club Bragging rights Badge of honour	No real sense of what the Raine Study is “all about”

Active participants invariably described the Raine Study as longstanding, internationally unique, and involving large numbers of participants. They highlighted its **credibility,** and their pride in what the Raine Study stood for:

I think it's something that Western Australia can be proud of as well because I remember always hearing it's one of the biggest, longest in the world…and it's respected for that. (Josie, active)

There's very few cohorts in the world ... where they've got data from before people were [born] as well as after. And then at 28-years, a follow-up study on 2,000 or something people… That's a very sizeable cohort… …that's very valuable. (Kate, active)

As well as active participants appreciating the **longevity** of the Study, they also emphasised the **effort** involved in the Raine Study, acknowledging managers must have “fought” hard to keep it going for so long.

Active participants saw themselves as *part* of the Raine Study **family**, describing themselves as “Raine Study babies” or “Raine Study kids”:

…it's weird, it's like you've been doing this thing your whole life and I guess it actually becomes part of your identity in a way... It's nice if you're out or you're getting to know someone and you find out that they're in the Raine Study too. I like that. (Cassie, active)

Being part of the Raine Study was described as “**cool**” by active participants; they said it differentiated them from others by affording them a unique story or talking point. *Being* studied was “unusual” and “uncommon” and made them part of a “cool little club”:

So being a Raine Study kid, growing up as a kid it was pretty cool because it was just something different, a fun fact about you and got you out of class sometimes and it was just - you got to go to cool places, see cool people. (Josie, active)

Active Raine Study participants appear to feel they are part of the brand, perhaps through the nostalgic links that were established through their early years:

So you know it’s one of those things that even as I grow older every now and then I’ll pop in and I’ll see somebody I recognise just because growing up you know you saw some of those people and you knew they were part of the study so it’s always been interesting in that regard. (Adrian, active)

Being in the Raine Study ‘club’ appeared to still give adult participants bragging rights among their peers. Having been ‘offered up’ to the study as babies by their parents gives them a ‘free pass’ to brag about their involvement and their participation earns them respect:

When I was telling my friends – I was out last night and we were talking about what we were doing over the weekend and I was saying, oh, going to the Raine Study. And my friends were, ‘oh what is that’ and then you explain the study, the significance of the study, all the things we’ve been able to contribute to as a group, that’s actually really cool… that's definitely the thing that makes you continue or makes me continue. (Cassie, active)

Active participants also seemed to view their familiarity with medical conventions as a **badge of honour** contributing to their identity as Raine Study participants. Having engaged in many different medical tests and processes, they had mastered what other people would generally fear. Some were proud that nothing they were asked to do as part of the Raine Study fazed them that much:

It's not often you meet someone that's gone through the same testing. If I have to go for a blood test say at the doctors, I've had so many already because of the Raine study but I'm just like yeah, whatever. And [when you] meet someone [and they ask] ‘did you ever do these tests’; I went yeah I did and you can just talk about the tests to other people. (Catriona, active)

In contrast, inactive participants did not describe any personal relationship, identification, or connection to the Raine Study ‘family’, suggesting it lacked a clear positioning or identity in their mind. Eight of the 12 inactive participants said they really did not know what the Raine Study was “all about”:

To be honest, I don't know – exactly know what the Raine Study is trying to achieve, the purpose of it. I really... I honestly don't know. (Richard, inactive)

All inactive participants described their experience as much like going to a doctor’s appointment every few years and expressed no affinity with the study or the brand. They appeared to feel the study had no impact on their lives apart from providing their yearly Raine Study birthday card. Inactive participants seemed somewhat confused by questions relating to branding, said they do not really think about it, and that it never comes up in conversation.

The next sections present the findings relating to the 4Ps and reveal additional critical insights to further enhance longer term positioning and branding of the Raine Study.

### Product

**Fig. 2:**
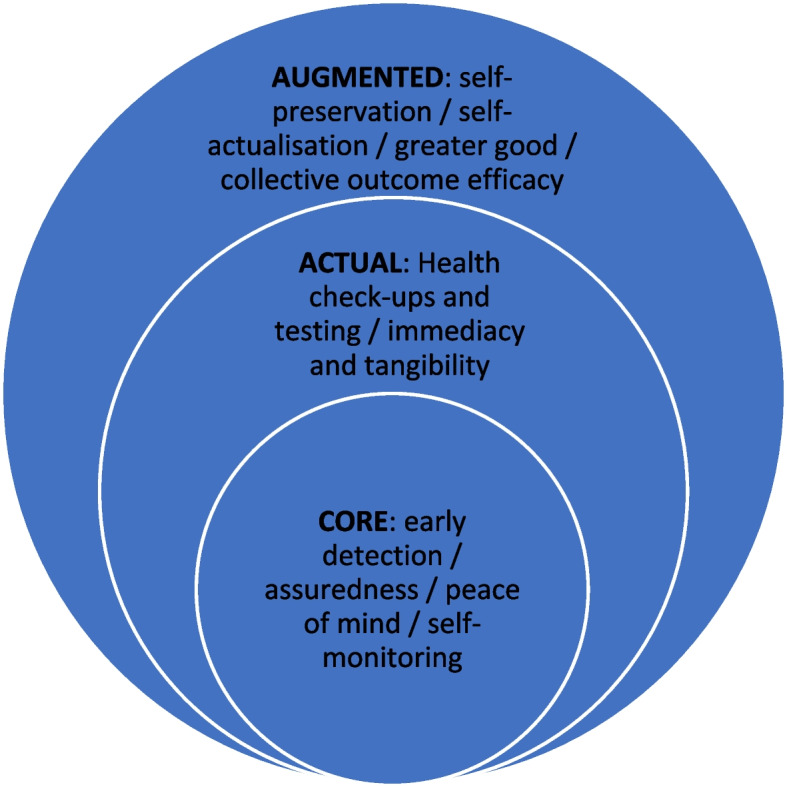
The core, actual and augmented product

The product component of the 4Ps refers to the benefits associated with participating in the Raine Study. Benefits of value to participants were classified as personal and collective in our first paper [15], and are reinterpreted here through the paradigm of the 4Ps. From the cohort’s perspective, the benefits are constructed as the core, actual and augmented components of a product offering. Data analysis identified themes of early detection of health issues, assuredness, peace of mind, and self-monitoring that appeared to empower Raine Study participants [15], and are therefore conceptualised as the core product (see Fig. 2). The actual product consists of the benefits that Raine Study participants received through regular health check-ups and testing, along with the immediacy and tangibility of the health information delivered. These actual products can be further enhanced or augmented when Raine Study participants draw on this information in the future (self-preservation) and to enhance their sense of self-actualisation. The most significant product augmentation is delivered through the collective benefits noted by Raine Study participants, particularly through the belief they were contributing to the greater good, and their efforts were making a difference for generations to come (collective outcome efficacy). Active participants perceived much more value from these benefits than the inactive participants [15], primarily because inactive participants experienced greater barriers to their participation.

Analysing the qualitative data further to reveal any gaps or enhancements applicable to the Raine Study product yielded an additional theme as listed in Table 3.

**Table 3:** Additional product insights

Theme	Subtheme	Codes	
		***Active participants***	***Non-active participants***
**Product**	Impacts and outcomes	Owning their data Wanting to know more ahead of time	No clear sense of purpose

One opportunity to leverage the product emerged when active participants discussed a desire to know more about the **impacts and outcomes** of *their* data, and how it was being used to advance medical knowledge. They alluded to the idea the studies conducted were often very broad or very vague and indicated they would really like to know what the end results were. Most were able to recall important studies about asthma, breast feeding and sleep, but still wanted to know more specifically, how their data was providing knowledge or benefit to others.

Although some active participants wanted to be sent specific information like journal articles or other publications, most were more interested in being kept informed through summarised reports directed at them as the ‘**owners**’ of the data. Many expressed a strong interest in receiving information that would enable them to really understand what the “big” results were. For example, Maree described how knowing the outcomes of these big studies – like the breast tissue study – could one day be very relevant to her.

Most of the inactive participants shared similar views about ‘wanting to know', despite lacking **any clear sense** of what the Raine Study was all about or what had been discovered since it began:

…it would be good to know…all the information put together, what have you found out or what do you now know which you didn't know 27, 28 years ago because of it. (Aaron, inactive)

These findings suggest that alerting participants to information to be released to the public or in a media release ahead of time might further enhance their connection to the brand, emphasising they are more than providers of data, reinforcing the respect bestowed upon participants, and adding value to their roles and their perception of the product. One active participant discussed her reaction to encountering news about the Raine Study in the public domain:

I'll see something like a supplement that's in the newspaper or some sort of segment on the news and think, oh it would have been nice to know about that before it went out, to feel like oh I'm a bit special, I'm a part of this, I'm seeing this before the public does. And I always feel like it would be nice if I could have seen that before it went out to the public, just to give me that special sense of I saw that before everyone else. (Pia, active)

Such findings relating to participants’ perception of the product, can be used in conjunction with the personal and collective benefits identified in our initial paper [15], to develop a stronger product concept for both active and inactive participants. Inactive participant Richard, whose life was very busy with work and family commitments, saw some value in a more personalised and tailored report benchmarking his health against others from the Raine Study:

If they could let us know how…how you're going compared to what the rest of - everyone else is doing. (Richard, inactive)

In particular, cohort managers could consider sending such reports out in the weeks before making requests for participants to attend their next appointments, as this might help to make the ‘product’ more ‘front of mind’ and ‘valuable’ for inactive participants.

### Price

The data were also analysed in terms of Price, encompassing the associated total costs (monetary and non-monetary) of the product offering to participants. As there are no direct monetary costs to participate in the Raine Study, the non-monetary costs and some minor monetary pricing benefits are depicted thematically in Table 4.

**Table 4:** Pricing insights

Theme	Subtheme	Codes	
		***Active participants***	***Non-active participants***
**Price**	Non-monetary costs	Lab rats (taken on the chin) Time / energy / effort Privacy risk Physical discomfort Psychological or emotional distress Pressure to perform	Lab rats (disgruntled)
	Monetary pricing benefits	Free check-ups/ free stuff	N/A

The first non-monetary theme relates to the participants’ role as ‘**lab rats’**, a term used by both active and inactive participants. The active participants seemed to simply accept this role, deriving such valuable benefits from their participation that the unpleasant aspects (e.g., pain from needles) were simply accepted as part of the process.:

…as much as I complain about it in my brain, to put on a coat, a lab coat, and just be a lab rat for the day, I come with the mindset - my mindset is just like ‘all right, they've got you for the day, let them do whatever they need to do’ and I just pretend I'm a specimen. (Cassie, active)

Active participants expressed some reservations about their roles as lab rats:

I think they've got to be careful to keep it - for me anyway to keep it health related. You don't want to feel like you're being used too much. And whilst I'm happy to contribute, we've had this economic survey and my head went, ‘how is this relevant?’ And I'm still quite happy to do it but I'm just thinking…I literally just came in for a DEXA scan and now I'm doing this, I don't see how this is relevant. ... if they keep bringing in these random topics that aren't necessarily health related or we've done before, I’m not sure that would be a good thing for some people. (Alison, active)

…they had researchers from overseas and stuff so obviously they’re using us because…they've got people here and I just wondered whether if they keep using us as a group of people to do research, if it starts to become too much or unrelated to what we've always done. I don't know if that's a good thing. (Catriona, active)

The active participants acknowledged the inconvenience associated with participation including the annoyances and frustrations in terms of completing surveys and questionnaires (time, energy, and effort), many of which they found to be long and laborious. They mostly soldiered on and completed these surveys despite finding it difficult to rationalise some of the questions:

I was agonising over not knowing because the answer [they were seeking] was different every week and I was just taking so long to answer […] it was taking forever. (Cassie, active)*.*

Some active participants expanded their comments to include concerns about **privacy,** considered here as a non-monetary cost or potential risk. While they generally understood that their data was secure, they acknowledged others’ concerns about survey questions on taboo topics such as drugs and drinking habits:

I think some people might be worried that maybe this information isn't confidential and what implications would that have for me if I'm a [professional] that's doing something and then I write down I'm smoking ice five times a week when I go home, what happens if my boss gets access to that information. Even though it wouldn't… (Maree, active)

Some active participants reported testing processes that made them feel physically or emotionally uncomfortable, thus potentially diminishing the core product (benefit) for these participants. Cassie described a particularly awkward test:

…they asked us to do a faeces sample. So, they gave us this pot to take home and you're meant to put it on your toilet and this whole thing and then send the specimen back in the mail. I had the pot and I couldn't do it. And they kept on sending me messages like… “Are you doing it?” And I said no, I'm sending the whole thing back empty because I was – [laughter]. And that's probably the only thing… I don't want to deal with that. Yeah, that was a bit too much. (Cassie, active)

Other unpleasant costs included psychological or emotional distress, with one active participant “really upset” as a child by a test requiring her to strip down to her underwear. Body composition tests, particularly tests done around other Raine Study kids, felt awkward to Renae when she was going through puberty:

…I hit puberty very young and so I think I got my period when I was eight or nine… I was feeling weird anyway but it wasn't very nice to be measured. And I remember you'd do it in a group of a few people. Other kids would be outside of the room or something and thinking oh I'm very different... …and that very awkward time that usually hits when you're in your early teens, it hit me five years earlier. And then they brought out a special questionnaire for me about my period and I was like well this is bullshit. But that was more because I already felt awkward and because I already felt like, different! That would be the only negative feelings I felt and that was just because I was an outlier. (Renae, active)

Several active participants described feeling anxious about a ‘stress test’ used to investigate hormonal stress response status. Kate experienced “social pressure” when asked to do the IQ tests, primarily because the questions were challenging, and she had no indication of how she was going:

…of course…they're particularly not allowed to give you feedback, but yeah, you don't know that when you're 18. I would probably feel exactly the same amount of pressure of ‘oh God, I'm stupid’, whenever being asked questions of - verbal questions of formal logic then you're like, ‘stop judging me’. (Kate, active)

While quick to defend the importance of the research, most of the active participants recalled experiences where they felt put under pressure or risked loss of face or being judged in some way. Kate described the pressure that children (in particular) might feel to perform during testings.

I think I probably messed up the data a little bit when ..I was 10, ... . I wasn't trying to mess with what they were doing; I was trying to be - I was trying to have a better heart rate result or something! (Kate, active)

Active participants felt pressure to ‘perform’ in order to achieve a good result for the study and for themselves, especially when it came to measurements of weight or muscle mass. Most active female participants disliked the DEXA scan because it showed whether they had put on weight. One active male participant delayed his appointment to have a scan measuring his body fat composition because he had put on weight, and regarded feeling self-conscious as one “unfortunate side effect” of the study:

… I sort of put it off in my head ‘cause I don’t really want to do that. I could be better. And that’s the really hard part, is I want to be my best for the study, but it’s supposed to be a representative, you know, population sample. So really you shouldn’t try and be you know the best. (Adrian, active)

Inactive participants appeared to resent being ‘lab rats’, mentioning how they felt “used”, and that they “couldn’t be bothered” expending the energy required to attend testings:

Sometimes feel a bit used - like a sci-fi experiment… Sometimes you feel like a lab rat. But it’s not their fault. Maybe they could fix that a bit. (Chris, inactive)

…it was probably the inconveniences of it and yeah, I suppose…just being poked and prodded. (Aaron, inactive)

Michaela questioned what ‘they’ might do (with her) in the future:

Feels like an experiment. Does anyone really appreciate that their data will always be out there? It feels a little strange. What if they can somehow clone me in the future? What do I get out of it? It’s a lot of effort really. (Michaela, inactive)

Inactive participants were more vocal about inconvenience versus benefit, and more likely to describe these types of psychological costs. One inactive participant “vaguely” recalled feeling awkward about being asked to participate in a fertility test that was a “bit personal” for his liking.

These comments demonstrate that inactive participants may resent being ‘used’ for science because they do not perceive sufficient personal benefit (“what’s in it for me”) to outweigh the costs or risks of participation. Given barriers around other socio-ecological forces are also considered (see 15), it is not surprising that these participants became inactive.

The data coded under pricing invariably revealed insights about the psychological, emotional, and at times, physical costs participants incurred as a result of their participation. However, some pricing benefits were noted by several active participants. Once again, these are closely linked to findings about personal benefits described in the initial paper [15], including early detection, peace of mind and self-monitoring. Examining these findings now from a pricing perspective provides an opportunity to leverage these benefits through a social marketing strategy, reducing some of the reported costs.

Noteworthy benefits for active participants described in the initial paper [15] related to receiving (free) health check-ups and screenings. Some active participants knew these tests would otherwise cost hundreds of dollars.

They put out in this call out, they mention that ‘hey the MRI you're getting is worth $700’. And I'm not a hypochondriac by any means but I'm like well I'd much rather get an MRI when people think there's nothing wrong with me and find out, hey, there's something wrong with me, then six months down the track find out there's something going odd with my body. So that in itself is a pretty great incentive. (Josie, active)

…finding out the information [from check-ups] is always nice, particularly with some of the stuff that you know would be expensive to seek privately. (Adrian, active)

Hence, pricing strategies should emphasise the total value of both monetary and non-monetary for participants. Active participants mentioned other ‘free stuff’ they were provided on testing days, fondly recalling receiving gift vouchers when they were kids, and enjoying free breakfast and cheese toasties at the end of their follow-up sessions. While these ‘freebies’ appear somewhat trivial, they may now be expected by participants. Participants can and do notice and resent the absence of expected small and trifling freebies. One inactive participant complained that the *only* free stuff she *ever* received was a T-shirt. It is therefore worth considering what the tipping point might be for active participants whose participation benefits are devalued or underdelivered.

### Place

In social marketing, ‘Place’ is where the target market accesses the social product on offer. While some research activities (e.g., completing surveys) were performed in the participants’ homes, the physical location of the Raine Study’s headquarters is the primary place that participants attend for follow-up testing. The current Raine Study House is sited on a bustling university campus, but has an intimate, family-type feel, rather than a clinical or institutional presence. The Raine Study had been located at a children’s research institution next to Perth’s children’s hospital for 20 years, and prior to that at a women’s hospital. While those venues were appropriate for the cohort as children, the current location may be more fitting for adult participants.

Our first paper identified barriers relating to place, with inactive participants finding ‘life gets in the way’ made it difficult to take time off work or give up family time to attend follow-up studies at Raine Study House [15]. This section draws attention to place-related issues by considering elements that might enhance a sense of place (see Table 5) and improve processes occurring while participants are at Raine Study House.

**Table 5:** Place insights

Theme	Subtheme	Codes	
		***Active participants***	***Non-active participants***
**Place**	On site processes	Positive environment	Positive environment
		Friendly and familiar staff	Friendly staff

Inactive participants suggested the Raine Study implement a telehealth service, or home visits [15]. One inactive participant recalled Raine Study staff coming to her when she was younger for aspects of the study, such as collecting bloods and picking up questionnaires.

…every time we had appointments and then if I couldn’t make it somewhere, they would come out so it was very accommodating, so I found that was really good. (Jayda, inactive)

Other participants suggested it would be easier to attend follow-ups if Raine Study appointments were undertaken in local hub-like centres, such as hospitals or other local health services, or via a mobile bus or van. Some of these more mobile-type strategies have been trialled in the past and are currently used where possible, but they remain big picture ideas requiring funds already in short supply.

Active participants spoke more about the supportive nature of the Raine Study processes, but both active and inactive participants described the Raine Study environment in positive terms:

They always just seemed so grateful for your participation and it was never like if you didn't want to do a blood sample or something you never felt like you were expected to, it always just felt like this very thankful environment. So, it was never, you have to do this. (Josie, active)

Inactive participants referred to the consistency of the testing processes. Despite the unpleasant costs described earlier, all the participants appeared satisfied with the study’s logistical arrangements. Although active participants were more likely to praise or commend the Raine Study for this, even inactive participants described their experiences in positive terms.

Most active participants praised the staff who made the Raine Study such a positive and supportive ‘place’. Cassie, an active participant, described the staff as “chirpy, nice and happy”, and explained that they “do their best to make it as fast and as pain-free as possible”. Even the inactive participants’ more cursory comments acknowledged how ‘nice’ the Raine Study staff were. One inactive participant fondly recalled Raine Study staff helping her through some difficult testing that she had initially been reluctant to do:

…there was one time I was, ‘I'm not going to be able to do it and she's just like let's just try, I'll do it with you’ and like yeah they were - I don't know, they were always just really great. Never made you feel uncomfortable or self-conscious, especially when I was younger as well, I wasn't very confident and so yeah, they never made me feel like I was forced into anything or uncomfortable in any way. (Simone, inactive)

Active participants were much more likely to name Raine Study staff or describe staff members who they had met regularly. The fact that the phlebotomist was “still the same lady” enabled Josie to develop confidence in her skills:

I still remember the phlebotomist, the woman who collects blood, she’d be the same woman basically forever! So, I saw her this morning and I said, you’re the blood lady because I remember there were a couple of times when we were teenagers that they wanted very early morning blood samples. …the first time they asked me for a blood sample I said no and I didn’t feel any judgement for that but the next time when I said yes I remember they came around 6:00 in the morning and I had to get out of bed and still the same lady! And she’s great, she takes blood better than – I had a bunch of blood tests this year and she said low iron and she’s got some very good blood taking skills. (Josie, active)

Participants’ trust in staff can help to reduce the considerable unpleasant cost associated with blood tests and other tests and surveys. Helping staff to get to know participants and build trusting relationships is very important for participants. The enthusiasm of the staff and their ability to communicate complex medical language were important for Josie and Maree:

…a lot of the other staff have been around long-term… I think one of the women this morning…who was going through the breast study thing with me, she's got a couple of different jobs. This is a bit of a random gig for her, but she's also doing it because of her interest in the area, so she's passionate and enthusiastic and that's all you really need: people who want to be there to do it and that's always been my experience. (Josie, active)

…the staff are always - they always have this super friendly attitude - they're still really knowledgeable and professional but just very relatable, I think, and I like how... so many people that are educated, they're speaking all this lingo but a lot of the general population maybe can't understand that and making it really relatable to their own lives and [Raine Study staff member] is really good at that. She was saying that that's what she likes to - she likes to really make it clear for people what this actually means, which is good. (Maree, active)

Active participants’ comfort and pride in their lab rat role may reflect the Raine Study communication culture of always treating them as more than mere lab rats:

It's built up over a lifetime of just not being exploited, not being treated badly or made to feel like a rat in a cage. I think my - yeah my experience is probably more on the more positive side of the average and maybe that's just because I just had really good people that I've always been tested by and spoken to a bit. (Renae, active)

The processes and people situated at Raine Study House enhance the Study’s overall product offering. Participants’ appreciation of staff respect and care for them throughout the study affirms that these affective attributes are key for participants physically present for testing. Other important place factors apply to the inactive participants who find it difficult to attend Raine Study House.

### Promotion

Promotion refers to the communication channels used to deliver key messages to the target market(s) in order to persuade them to adopt or perform the desired behaviour. As effective promotional strategies could support and reinforce the Raine Study brand, this study identified gaps or opportunities to persuade participants to keep coming back for follow-up tests. Themes relating to Promotion are listed in Table 6 below.

**Table 6:** Promotional insights

Theme	Subtheme	Codes	
		***Active participants***	***Non-active participants***
**Promotion**	The Family brand	Events Birthday cards Untapped social media Building inter-participant relationships	Birthday cards Untapped social media – difficult topics Being real

For Renae, the “marketing” of the Raine Study, at least early on, was integral to the positioned meaning of the Raine Study:

So earlier on the communications department was quite good. I think the marketing of Raine Study was quite good and I remember when I was younger, when I was a teenager there was this real emphasis on the Raine family. They hosted a big birthday party for us and there was this - there was a Facebook page and it made you feel like you were a member (the Raine family). That's dropped off a little bit now. (Renae, active)

As discussed earlier, the ‘Raine Study family’ really resonated with active participants. Finding new ways to reinforce this or strategies to re-position the family orientation (for inactive participants), is a valuable area to explore. While e**vents** such as the birthday party could enhance this perception, other promotional channels might prove to be more effective given the busyness of participants’ adult lives.

In particular, although the Raine Study has used Facebook intermittently to communicate with participants since 2008, most participants saw platforms like Facebook as underutilised tools for ‘reaching’ them:

There was a Facebook group for a while and I think that's dropped off as well, where people would message each other. I just remember thinking there's a disparate group of people all around Perth that I happen to have a similar age in common with but I also have this very distinct shared experience. (Renae, active)

Yeah, it's relatively untapped […] it doesn't get the publicity or the promotion that it deserves. I think they definitely need to up their social media presence. (Simone, inactive)

I suppose as social media changed […] it makes it even easier to post things and let people know what's going on. Maybe updates…that's what Facebook's for, you update on things, but it'd be good to see things that are happening more regularly. (Aaron, inactive)

Aaron also saw value in using Facebook to call for volunteers to participate in upcoming studies. Derek (also inactive) suggested posts of this nature promoting interesting studies on offer could attract his attention.

Given inactive participants had very little understanding of what the Raine Study is all about, and both active and inactive participants wanted to know more about the outcomes or impacts of the studies, Raine Study managers could enhance their efforts to promote engagement, understanding and outcomes. If even active participants lose sight of the study’s purpose, position and brand, the risk of attrition soars. Using channels like Facebook to promote, translate and disseminate study outcomes and impacts can support the product offering and build a strong brand position.

Given the potential to erode or enhance the sense of community or the family-like presence that the Raine Study should continue to communicate, effective use of social media channels such as Facebook requires active and sensitive moderation to carefully manage public discussions in ways that reinforce public health messages without alienating members. In public forums, it may be “self-defeating to restrict topics of interest to members” ( [22] p. 72). Dealing with ‘fake news’ posted on public forums remains a challenging issue for health organisations trying to maintain a trustworthy brand and promote health literacy in a respectful way. Equally, it is important not to endorse or support incorrect views that ignore evidence or that have potentially damaging effects on others.

One inactive participant recounted observing a potentially corrosive incident on the Raine Study Facebook group. Aaron recalled a Raine Study participant posting a comment that could be interpreted as supporting an anti-health movement and then making a very diplomatic and genuine request for the Raine Study managers to comment on the science of the issue being debated. He recalled this conversation being shut down and the Facebook poster’s subsequent alienation from the group. Raine Study staff had provided evidence-based information in response to the post and spoken with the poster to try and resolve any potential fallout.

Participants still appreciated more traditional communication channels. All active and inactive participants mentioned receiving birthday cards, which help to reinforce the family-like nature of the Raine Study brand:

Yeah, and then also that we get birthday cards and yeah they're printed out and they're mass printed and you can tell there's nothing. But it's still a nice little touch. (Renae, active)

…it's not like you wait for it to come in your letterbox but it's nice to know that they care about you for more than just the research. (Maree, active)

Some active Raine Study participants offered promotional-type suggestions to further enhance the ‘Raine family’ brand. As Raine Study participants were “all of the same vintage”, they saw opportunities to celebrate who they are as people by using dating sites, a Raine Study ‘Survivor’ series or perhaps even a new ‘Bachelor’ series, cooking classes and long-table banquets to promote and enhance the Raine Study brand. Active participants were excited by the idea they were a special group, and that celebrations of Raine Study participation could resemble high school reunions. A few participants thought that novelty events would be highly appropriate for their age demographic.

By creating opportunities to build inter-participant relationships or a sense of community, those novel concepts could help to promote the Raine Study brand, at least for active participants. While this paper described how (non-health related) studies or tests which participants are asked to undertake during follow-ups could be a tipping point for attrition, further research could determine whether participants desire engagement not related to follow-up attendance. Participant reports of feeling used suggest brand connection could be enhanced by creating more meaningful relationships or providing some type of escape [23] from the busyness of their lives.

Feel a bit used at times because they send so many long surveys. I kinda wish they would do something interesting or fun. Perhaps about who we are or something about our personalities. (Richard, inactive)

For inactive participants who already see less value in the core product or feel the costs and barriers are too high, such novel activities may not, on their own, be strong enough to enhance participation.

## Discussion

This paper uses a framework based on the 4Ps of social marketing to elucidate important insights regarding attrition and retention in a longitudinal cohort study. Interpreting participant interviews through this framework emphasises the importance of implementing retention strategies addressing the different needs and preferences of active and non-active participants.

Our approach departs from most prior research on retention that lack a strong theoretical framework and focus on strategies summarised in Table 1. Strategies used in the Raine Study include friendly personnel and nonfinancial incentives and other benefits of the research. This study’s novel theoretical approach confirmed potential strategies, while significantly adding to knowledge about when and for who these strategies should be adopted.

This process of audience segmentation is a central feature of the social marketing framework and is used to understand and respond to different needs and preferences within the target population [13]. Our study indicated that Raine Study managers can use knowledge about how the 4Ps differ between active and non-active participants to tailor their efforts to reduce attrition and improve retention. For example, active participants perceived more value from the benefits of the product than inactive participants who experienced greater barriers to their participation. Thus, finding ways to enhance the product offering and reduce the costs for inactive participants is critical.

In addition, both active and inactive participants wanted to know more about the various research activities they had participated in. Active participants wanted to know more about the impacts and outcomes of their data and how it is being used to advance medical knowledge. Inactive participants wanted to know more about what the Raine Study was all about, and what the study had discovered since it began. Finding ways to communicate the relevance and importance of the performed studies to the entire cohort should therefore be prioritised.

Regarding Price, inactive participants identified several non-monetary costs, and in contrast to active participants, did not perceive any pricing benefits. Pricing strategies directed at inactive participants should emphasise the total (monetary and non-monetary) value of the benefits for participants. Active participants also perceived several non-monetary costs so managers need to ensure the perceived costs do not outweigh the perceived benefits for participants.

Participants who feel more autonomous [24] may also be more likely to keep coming back.

The notion of fostering a more meaningful level of engagement relates to a key philosophy in public health research that emphasises the importance of participants being involved throughout the research process, and not just consulted after the research has been decided [25]. Managers should therefore engage participants in meaningful discussions about the types of studies being planned and what participants might be asked to do, to ensure that what they are being asked to do is both reasonable and relevant. Although the Raine Study has established participant-led advisory committees, finding ways to communicate the relevance and importance of such studies to the wider cohort should be prioritised, especially if participants begin to question the legitimacy of their contribution as participants. Adopting this approach in a more systematic way for active participants could help the Raine Study ameliorate non-monetary costs and reduce potential attrition.

Place-related elements enhance a sense of place or processes that occur when participants are on site. Participants’ appreciation of the staff’s respect and care throughout the study indicates the Raine Study’s positive environment and friendly staff enhance its overall product offering.

Understanding Promotion enables study managers to determine any gaps or opportunities in communication channels that can be used to deliver key messages and retain participants for follow-up tests. Both active and inactive participants valued personal touches like birthday cards and agreed on the untapped potential of social media as a tool to improve attrition and retention. Developing a more active social media presence may aid the Raine Study to maintain ongoing contact between researchers and participants, develop relationships and increase participants’ commitment and engagement with the research [26, 27]. Leveraging the Raine Study’s social media presence is not without risk, but engaging participants through regular updates on study outcomes could enhance active participants’ identification as part of the Raine ‘family’ and strengthen their sense of brand ownership. Social media updates keeping the Raine Study ‘front of mind’ among inactive participants could foster stronger familiarity and ‘brand’ recognition.

Applying a social marketing framework to the management of cohort studies will ensure a comprehensive and tailored suite of strategies to aid retention, while ensuring adjustments to protocols are reflected in the literature will aid future research.

Some important study limitations should be considered before applying our findings to other studies. The 30+ year old Raine Study is located within the social, political and geographical context of an affluent state with a stable government, a mild climate and high-quality education and health services provided to all. Participants’ biases around participating in such a study and the costs they perceive in their ongoing participation may differ greatly from those in other circumstances. The long term and repeated engagement of participants in the Raine Study may result in brand positioning and price outcomes quite different to those for a shorter-term cohort. The perceptions of the young adult Generation 2 may differ from those of the original cohort participants known as Generation 1, who are now in middle age. Finally, our findings are limited to the perspectives of active and inactive participants who agreed to be interviewed.

## Conclusions

Social marketing offers a unique perspective on research participants in longitudinal studies as consumers or customers. Conceptualising participation in such studies as an ‘exchange’ reveals opportunities for cohort managers and researchers to develop a more mutually beneficial product, thus building a greater sense of connection and ownership among participants. Since the quality of longitudinal studies relies on retention of a sufficient and unbiased sample, understanding how participants view their role from a consumer perspective can be critical to enhancing ‘brand loyalty’.

Our research innovatively applied a social marketing framework to explore issues related to engagement and retention among participants in the Raine Study, a long-running cohort study. Interpreting participants’ experiences through the social marketing ‘mix’ of Product, Price, Place and Promotion enabled a deeper understanding of how active and inactive participants perceived the value and benefits accrued through their participation (the Product), relative to the costs they incurred (the Price). Profiling the Raine Study cohort into specific segments enables development of differentiated product, place, pricing and promotional strategies targeted more precisely for cohort retention.

While Raine Study data collection typically focuses on one specific generation, providing a natural market segmentation, our findings suggest active and inactive cohort members constitute different target segments. The distinct differences in their relations to product and price factors indicates building brand meaning will likely require different strategies for active and inactive participants.

Active participants understood the purpose, prestige and importance of the Raine Study and demonstrated a strong affinity with the Raine Study brand. Implementing strategies informed by the 4Ps could help re-position and build the Raine Study brand, particularly for inactive participants. Findings relevant to Place and Promotion highlighted the potential value, particularly for inactive participants, in reinforcing the ‘big idea’ of the ‘Raine Study Family’. Targeted social marketing strategies could leverage active participants’ commitment and further strengthen their identification with the Study ‘brand’, while addressing inactive participants’ perceptions that the price outweighs any product benefits.

The differences and similarities between active and inactive participants warrant further investigation and the trialling of specific strategies, perhaps through an RCT design. Demographic characteristics such as employment hours, family structure and commute times could reveal smaller segments relating to specific barriers, along with other psychographic and geographic parameters for both active and inactive participants.

As our research demonstrated, a social marketing approach yields unique and important insights into the research participant experience. To ensure authentic brand meaning is established and retained, managers of cohort studies should carefully co-create branding strategies with their participants. Brand managers must listen to the meanings that internal and external stakeholders attach to their brands, as interpreting that meaning is imperative for successful co-creation [28], and can be used to minimise attrition.

## Supplementary Information

**Additional file 1.** Interview Guide.

## Data Availability

The datasets generated and analysed during the current study are not publicly available due to confidentiality requirements specified by Edith Cowan University’s Human Research Ethics Committee. For more information about the data generated in this study, please contact the first author by email (l.costello@ecu.edu.au).

## Abbreviations

4Ps: refers to Product, Price, Place and Promotion in marketing theory
