# Supplementary material for: Applying the 4Ps of social marketing to retain and engage participants in longitudinal cohort studies: generation 2 Raine study participant perspectives
Source: BMC Med Res Methodol. 2022 Nov 5;22:288. doi: 10.1186/s12874-022-01778-4 (PMC9636764; doi:10.1186/s12874-022-01778-4)
Supplement: Supplementary file 1 — Additional file 1. Interview Guide. [file 12874_2022_1778_MOESM1_ESM.doc]

**Additional File 1: Semi-structured Interview Guide**

- 1. What has it been like being part of the Raine Study?
- Can you think of any particularly memorable moments as a participant?
- What do you like about being part of the Raine Study?
- Are there things you don’t like about the Raine Study?
  1. Can you recall what it was like when you were younger?
     - Did you get a chance to ask questions and offer your opinion about the research?
  2. Can you remember the first time you went to a follow-up without your parent or when you came of age?
  3. How have things changed now that you’re older?
  4. What about your life now? Are there things which make it easy or difficult to remain involved with the Raine Study?
  5. Do you have any suggestions for improving your involvement in the Raine Study? During follow-ups or in between? Which might make it easier for you to stay involved?
  6. What do you think makes people drop out of studies like Raine?
     - Would you ever withdraw? Why might that be? Have you ever been close to withdrawing?
  7. Why do you think people stay involved?
     - How about you? Why do you think you’ve stayed involved?
  8. Do you see yourself taking part for another 25 years? How long might you continue for?
  9. What does the Raine Study mean to you personally? How does it make you feel about yourself or your place in the world?
     - Do you ever talk about it with other people or friends? (rapport required: potentially personal/intrusive)
  10. How would you feel if you had to withdraw from the study, or if, for some reason, the study came to an end in 2 years?

How much do you think participants should be involved in making decisions about the Raine Study?

- Is there anything about which you think participants should have a say?
  1. What do you think are some good ways to make participants like yourself feel more connected to the Raine Study?
     - What do you think are good ways to engage children, teens and young adults in studies like Raine?
  2. Can you describe a time when you felt especially engaged in the Raine study?
- How has your sense of engagement changed over time?
  1. Have you ever felt somewhat or completely disengaged with the study and/or other participants or staff?
     - If so, what brought you ‘back’ to the study?
  2. Has anything ever gone wrong when you’ve attended follow-up?
     - Any procedures, tests, questionnaires etc..
  3. Is there anything that has particularly worried or concerned you about the Raine Study? Made you feel uncomfortable?
  4. How do you feel about other Raine participants/staff – what are relationships like between participants / between staff?
     - Have any friendships formed?
     - Are there any outside meet ups / events?
  5. If you could sum up your experience as a Raine participant so far, what might you say?
  6. Why might you recommend others to be part of a study like the Raine Study?
  7. If you were asked to promote participation in a new study (like Raine) what would you say to potential participants?
  8. Is there anything that has popped into your mind as we were talking about your experiences that we didn’t get to talk about?
